# From Mechanism to Management: The Link Between Inflammatory Bowel Disease and Cardiovascular Disease

**DOI:** 10.1002/iid3.70570

**Published:** 2026-11-10

**Authors:** Farah Yasmin, Mansoor Ahmed, Taha Bin Iftikhar, Asad ur Rab, Muhammad Saeed Qazi, Muhammad Sohaib Asghar, Mohammed Mahmmoud Fadelallah Eljack, Krutarth Pandya, M. Chadi Alraies

**Affiliations:** ^1^ Department of Internal Medicine Yale New Haven Health New Haven Connecticut USA; ^2^ Department of Internal Medicine Liaquat University of Medical & Health Sciences Jamshoro Pakistan; ^3^ Department of Internal Medicine Rawalpindi Medical University Rawalpindi Pakistan; ^4^ Department of Internal Medicine Foundation University Medical College Islamabad Pakistan; ^5^ Department of Internal Medicine Bilawal Medical College Jamshoro Pakistan; ^6^ Department of Internal Medicine AdventHealth Sebring Florida USA; ^7^ University of Bakhtalruda, Faculty of Medicine and Health Sciences Ad Douiem Sudan; ^8^ Department of Hospital Medicine Cleveland Clinic Cleveland Ohio USA; ^9^ Department of Cardiovascular Medicine, Detroit Medical Center DMC Heart Hospital Detroit Michigan USA

**Keywords:** arterial thromboembolism, coronary artery disease, inflammatory bowel disease

## Abstract

**Introduction:**

Inflammatory bowel diseases (IBDs) constituting the ulcerative colitis (UC) and Crohn's disease (CD) primarily affect the gastrointestinal tract and have been associated with the development of cardiovascular disease (CVDs). To strengthen this further, the focus of our narrative review is to highlight the clinical significance of understanding this association.

**Methods:**

We conducted a narrative review to discuss the mechanisms of pathogenesis underlying the link between the management of inflammatory bowel disease and cardiovascular disease.

**Discussion:**

The pathogenesis of the link likely involves dysregulated immune responses leading to overactivation of pro‐inflammatory cytokines, suggesting a shared inflammatory pathway. Both diseases share common risk factors such as obesity, diabetes, and gender. Genetic factors, including mutations in NOD2 and CDKN2A, also play a role in the overlap between IBDs and CVDs. Despite the recent developments, conventional cardiovascular risk assessment tools may not fully capture the potential elevated cardiovascular threat in IBD patients.

**Conclusion:**

Thus, proactive investigation with advanced diagnostic tools such as coronary artery calcium (CAC) scoring is crucial in reducing cardiovascular morbidity and mortality in these patients. Nevertheless, future research is needed to further clarify disease activity and its correlations.

**Clinical Trials Registration:**

NA.

## 1 Introduction

Inflammatory bowel diseases (IBDs), which include ulcerative colitis (UC) and Crohn's disease (CD), are characterized by chronic inflammation of the intestine primarily resulting from immune system dysregulation [1]. The 2019 Global Burden of Disease study estimated approximately 4.9 million cases of IBD worldwide, with the highest prevalence observed in China, accounting for 762,890 cases. Despite a decline in the global age‐standardized prevalence rate from 73.23 per 100,000 individuals in 1990 to 59.25 per 100,000, the mortality associated with IBDs continues to rise [2]. The potential drivers of this rising mortality include disease mechanisms such as complications or systemic inflammation. Furthermore, IBDs are linked to various extraintestinal manifestations, including musculoskeletal, dermatologic, renal, and cardiovascular complications [1]. Cardiovascular complications are among the more understudied or underrecognized outcomes. Considering that cardiovascular diseases (CVDs) represent the leading cause of morbidity and mortality on a global scale [3], recent research has identified an elevated risk of CVDs among patients with IBDs. Notably, this includes an increased incidence of coronary artery disease (CAD) and venous and arterial thromboembolism (VTE), highlighting the need for further investigation in this area [4, 5].

## 2 Literature Search and Selection

For this narrative review, the literature was identified using PubMed/MEDLINE and Google Scholar with combinations of the terms “inflammatory bowel disease,” “Crohn's disease,” “ulcerative colitis,” “cardiovascular disease,” “atherosclerosis,” “thromboembolism,” “endothelial dysfunction,” “gut microbiota,” “diabetes,” and related cardiovascular outcomes and therapies. Priority was given to systematic reviews, meta‐analyses, large observational cohorts, mechanistic studies, and recent clinically relevant publications. Articles were selected based on relevance to the relationship between IBD and CVD, with additional studies identified from reference lists of key publications. As this was a narrative review, no formal systematic‐review protocol or formal risk‐of‐bias assessment was undertaken; therefore, selection and publication bias cannot be excluded.

## 3 Shared Pathophysiological Mechanisms Linking IBD and CVD

The extraintestinal manifestations of IBDs stem from their pathogenetic mechanism, which is complex and still not completely understood [1]. Intestinal innate immunity plays a key role in the development of these diseases. It is primarily driven by cells like neutrophils, macrophages, and dendritic cells, which maintain the balance between defense against microorganisms and beneficial gut microbiota. In IBD, there is a dysregulation of this equilibrium, which causes overactivation of immune cells and results in a surge of pro‐inflammatory cytokines causing chronic intestinal inflammation [1]. This overactivation can lead to tissue damage, leading to the start and progression of the disease [6]. Interestingly, CVD is also related to inflammatory cells like macrophages, which lead to inflammation and fat deposition, a primary mechanism behind the development of atherosclerosis [7].

The available evidence supports a biologically plausible overlap between IBD and CVD, but the strength of evidence differs across proposed mechanisms. Inflammation, endothelial dysfunction, and altered gut microbial signaling are supported by clinical and experimental observations; however, most human data are observational and demonstrate associations rather than definitive causal relationships. These mechanisms may interact rather than operate independently: intestinal immune activation may increase systemic inflammatory signaling, while endothelial and metabolic disturbances may amplify vascular risk. Thus, the relationship is best viewed as a multifactorial inflammatory and metabolic network rather than a single causal pathway.

The shared pathophysiological mechanisms of these diseases have been illustrated in Figure 1.

**Figure 1:**
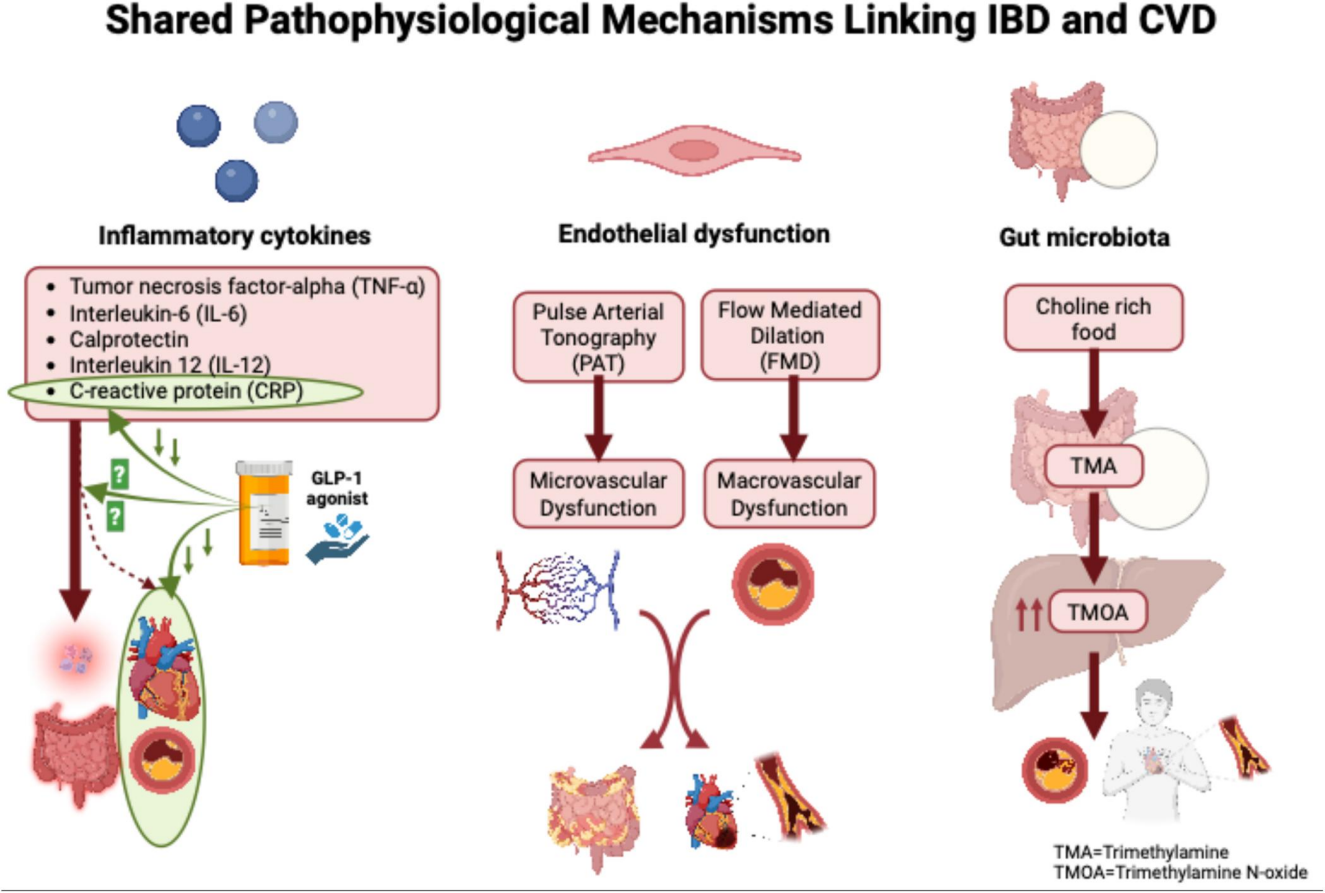
Shared pathophysiological mechanisms between IBD and CVD.

### 3.1 Inflammatory Cytokines

A variety of proinflammatory cytokines such as C‐reactive protein (CRP), tumor necrosis factor‐alpha (TNF‐α), interleukin‐6 (IL‐6), calprotectin, and interleukin 12 (IL‐12) are involved in the pathogenesis of IBD [8]. These biomarkers are also associated with CVD [7]. CRP has demonstrated predictive value as a marker for CVD risk [8]. Moreover, anti‐inflammatory pharmacological interventions like glucagon‐like peptide‐1 (GLP‐1) agonists have also shown effects in decreasing CVD risk and CRP levels [9]. Similarly, TNF‐α, IL‐6, and IL‐12 primarily regulate atherosclerosis and IBD [7, 8]. Calprotectin, a protein secreted by activated neutrophils, has also recently shown its association with an increase in risk for CVD events [10].

Collectively, these findings suggest that inflammatory biomarkers may reflect pathways shared by IBD and atherosclerosis, but biomarker associations should not be interpreted as proof that a specific cytokine directly causes cardiovascular events in IBD. Differences in disease activity, treatment exposure, comorbidities, and study populations may also contribute to the observed associations.

### 3.2 Endothelial Dysfunction

Endothelial dysfunction also plays an essential role in the development of atherosclerosis and vascular disease [11]. This dysfunction is also seen in patients with IBD and involves both microvascular and macrovascular dysfunction [12]. This has been demonstrated with reduced pulse arterial tonometry (PAT) indices and flow‐mediated dilation (FMD) levels, which serve as measuring scales for microvascular and macrovascular dysfunction, respectively [8]. Roifman et al. [12] assessed endothelial dysfunction and found that 48 patients with UC and CD had lower PAT and shear stress reactive hyperemia as compared to controls, which indicates endothelial dysfunction. Additionally, elevated levels of thrombomodulin in UC and increased inflammation of bowel tissue further indicate endothelial dysfunction [8].

The clinical relevance of endothelial dysfunction is therefore plausible but remains incompletely established. Findings from vascular‐function studies provide mechanistic support, whereas prospective evidence linking these measurements to hard cardiovascular outcomes in IBD remains limited. This distinction is important because endothelial dysfunction may be a marker of systemic inflammatory burden rather than an independent causal mediator.

### 3.3 Gut Microbiota

Alterations in gut microbiota are associated with both IBDs and CVDs. Intestinal dysbiosis, an imbalance in the gut microbiota, is a significant risk factor for CVDs [13]. It has been linked to the development of obesity and diabetes, conditions that increase the likelihood of CVD [14]. A recent cohort showed that circulating DNA (circDNA) in the plasma of CVD patients (median, 1925.64 ng/mL; IQR  =  391.83, 5395.92) was significantly higher than in healthy individuals (median, 37.85 ng/mL; IQR  =  32.80, 69.38) [15]. On the other hand, gut dysbiosis has been extensively studied as a key contributor to the pathogenesis of IBD [8]. Another dimension to this interplay is the synthesis of trimethylamine N‐oxide (TMAO) and its relation to CVD risk. Specifically, dietary precursors like choline are metabolized by gut bacteria into trimethylamine (TMA), which is subsequently converted into TMAO in the liver. High levels of TMAO have been associated with an increased risk of CVD [14, 16].

The gut–vascular relationship may also involve metabolic and microbial signaling, including TMAO production, intestinal permeability, and downstream inflammatory pathways. Nevertheless, current evidence largely demonstrates associations between dysbiosis or microbial metabolites and cardiovascular risk, and it remains uncertain whether these alterations are causes, consequences, or amplifiers of IBD and CVD. These mechanisms should therefore be considered complementary hypotheses rather than established causal pathways.

## 4 Genetic Linkage Between CVD and IBD

The role of inflammation has been pivotal in the development of CVD and IBD. Mutations in genes like nucleotide‐binding oligomerization domain 2 (NOD2), cyclin‐dependent kinase inhibitor 2A (CDKN2A), stromelysin‐1, and apolipoprotein E (ApoE) have been implicated in the pathogenesis of both IBD and CVD and may explain the linkage between the two [17]. Stronger genetic linkage exists for CD as compared to UC [18].

The genetic link between CVD and IBD has been summarized in Figure 2.

**Figure 2:**
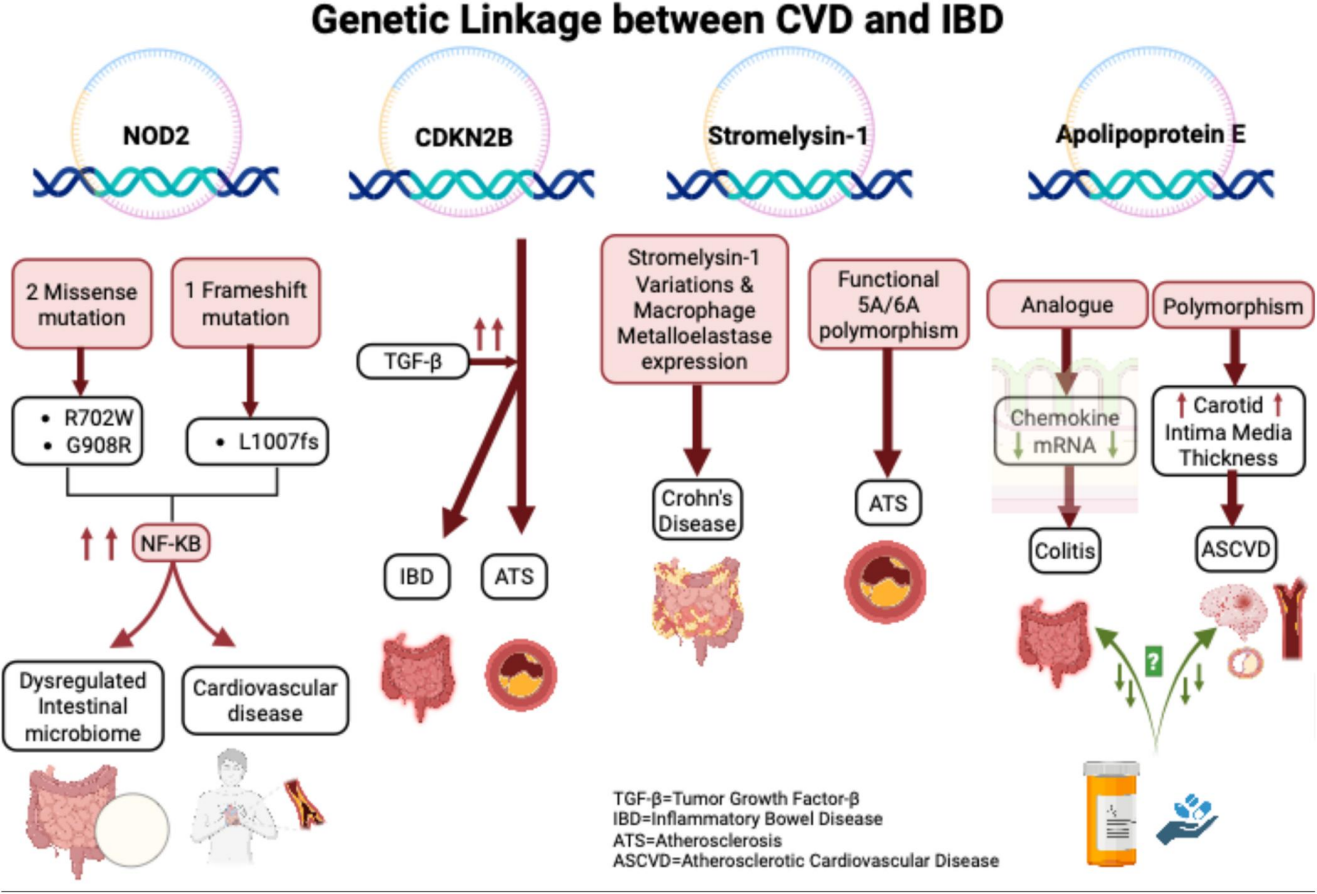
Genetic linkage between IBD and CVD.

### 4.1 NOD2 Gene

NOD 2 Polymorphism was implicated in CAD [19]. NOD2 was the first gene identified as a risk factor for CD [20]. The three main CD‐associated NOD2 variants include one frameshift mutation (L1007fs) and two missense mutations (R702W and G908R), which have been implicated in inappropriate NF‐κB activation and impaired recognition of microbial components, resulting in dysregulation of the intestinal microbiome [21].

### 4.2 CDKN2B Gene

CDKN2B is induced by transforming growth factor beta (TGF‐β), and the TGF‐β signaling system is implicated in the pathogenesis of atherosclerosis and IBD [22]. A common sequence variant on chromosome 9p21 is associated with a higher risk of myocardial infarction (MI) [23]. The identified variant, adjacent to tumor suppressor genes CDKN2A and CDKN2B, was associated with the IBD with high significance [24].

### 4.3 Stromelysin‐1

Functional 5A/6A polymorphism of the stromelysin‐1 promoter has been implicated as a potential genetic marker for the progression of angiographically determined atherosclerosis in patients with CAD [25]. Recently, a novel IL‐6 gene functional G/C polymorphism at position 2174 in the promoter region has also been reported [26]. Variations in Stromelysin‐1 and macrophage metalloelastase expression in the intestinal mucosa of Crohn's disease patients were identified [27].

### 4.4 Apolipoprotein E

Genetic polymorphisms in apolipoprotein E are associated with carotid intima‐media thickness leading to ASCVD [28]. Apart from its classical role in lipid metabolism, an immunomodulatory role for apolipoprotein E has also been described. An apolipoprotein E analog has been shown to downregulate chemokine messenger RNA (mRNA) expression on intestinal epithelial cells from mice treated with dextran sodium sulfate to induce experimental colitis [29].

This implies that inflammation‐driven genetic pathways may serve as therapeutic targets for both CVD and IBD, allowing for potential crossover treatments. Additionally, individuals with genetic predispositions to one disease may be at an increased risk for the other, emphasizing the need for integrated screening and management strategies.

## 5 Cardiovascular Manifestations in IBD

While reviewing epidemiological data, both the increased cardiovascular risk in patients with IBD and the emerging evidence of elevated IBD risk among cardiovascular patients were profound. IBD has not been established as an independent risk factor for myocarditis, pericarditis, and heart failure, but evidence from large cohort studies has demonstrated an increased risk of hospitalization for heart failure among IBD patients, particularly during periods of active disease [30], and a sustained elevated risk of heart failure for over two decades following IBD diagnosis [31]. Furthermore, while the increased CV risk in IBD patients is well recognized, the reciprocal association and the heightened risk of IBD among cardiovascular patients are supported by recently published data [32], which could serve as a foundation for a more integrative discussion of shared inflammatory pathways and immune mechanisms that underlie both disease states.

### 5.1 Coronary and Peripheral Arterial Disease

Studies have shown that IBD patients tend to have a higher risk of CAD and PAD. A cross‐sectional study in 2013 showed that subjects with IBD experienced atherosclerotic cardiovascular disease (ASCVD) events more often compared with matched controls [odds ratio (OR) 2.01, 95% confidence interval (CI) 1.23–3.27], specifically heart failure and coronary heart disease [33]. Similarly, a nationwide population‐based cohort study demonstrated that the risk of developing PAD was 1.29‐fold in patients with IBD compared with the comparison cohort [34]. Additionally, a UK‐based cohort also revealed an increased risk in the younger population of CD to develop ischemic stroke [35]. This correlation is mainly possible due to a similar mechanism of high inflammatory load in the intestinal and vessel walls, which drives the progression of atherosclerosis, subsequently resulting in plaque rupture and thrombosis [8].

These associations are likely driven by the chronic inflammatory burden observed in IBD, which promotes the progression of atherosclerosis and increases susceptibility to plaque instability, rupture, and thrombosis [8]. Accelerated atherosclerosis has been consistently observed in patients with IBD and is thought to contribute to the higher incidence of arterial thromboembolic events, including acute myocardial infarction and ischemic stroke [36]. However, it is important to note that some studies have questioned the strength of this association, highlighting inconsistencies across populations and study designs [37].

### 5.2 Arterial and Venous Thromboembolism

IBD is linked to an increased risk of arterial and venous thromboembolism (VTE). Studies have shown that IBD patients have a higher incidence of acute arterial thrombotic events and VTE, including DVT and pulmonary embolism (PE) [36]. The increased risk occurs due to hypercoagulability that is seen in IBD [36]. A meta‐analysis of 207,814 IBD patients concluded that overall IBD was associated with an increased risk of VTE (RR, 1.96; 95% CI, 1.67–2.30). There was also a statistically significant increased risk of developing DVT and PE associated with IBD [37].

### 5.3 Heart Failure

Unlike similar inflammatory conditions, for example rheumatoid arthritis and systemic lupus erythematosus, IBD has not been established as an independent risk factor for myocarditis, pericarditis, and heart failure. Some studies have found an association between IBD and heart failure in female patients with UC, and those diagnosed after 40 are at greatest risk [38]; however, declining estrogenic effects in this cohort can question the validity of this association. The risk of heart failure has also been associated with steroid use in patients with IBD [38].

### 5.4 Atrial Fibrillation

The prevalence of atrial fibrillation has been noted to be many times higher than in the general population in IBD patients, as seen in multiple studies [39, 40]. This pattern carries across all age groups and is present in patients with both UC and CD. The mechanism, though not well understood, is related to the baseline inflammatory state of the body. A higher rate of atrial fibrillation was observed in patients with active flares as compared to those with IBD activity. The increased prevalence across all age groups is compelling and highlights the systemic impact of inflammation. This may support the role of routine rhythm monitoring in high‐risk IBD patients [41, 42].

## 6 Shared Risk Factors Linking IBD With CVD

### 6.1 Smoking

Smoking is a well‐established and modifiable risk factor for ASCVD, but it is also associated with an increased risk of clinical recurrence of CD. A cohort in 2014 by Dregan et al. [43] concluded that patients with CD tended to smoke more than patients with UC and controls. In a retrospective series of 182 CD patients, smoking was found to be the only significant risk factor associated with clinical and surgical recurrence of disease [21]. Similarly, a cohort of 700 IBD patients [44] showed that smokers with UC had a higher rate of new cancer emergence (16% vs. 6.7%, *p* < 0.05) and mortality (16% vs. 4.9%, *p* < 0.01) in comparison to the nonsmokers group.

### 6.2 Diabetes

The association between diabetes and CVD is well‐established [45]. However, the association of IBD with diabetes requires more research. However, there is some data to suggest that there may be a possible association. In 2019, in a nationwide cohort of 8070 patients with IBD [46] who were followed up for 5 years, it was concluded that the risk of diabetes was significantly higher if patients had CD (HR, 1.677; 95% CI, 1.408–1.997), but not if patients had UC (HR, 1.061; 95% CI, 0.973–1.156). Thus, regular monitoring of glucose should be considered in patients with CD. Additional research is required.

Recent evidence further supports the clinical importance of metabolic comorbidity in Crohn's disease. In a real‐world propensity‐score‐matched analysis of 7182 patients with Crohn's disease and type 2 diabetes mellitus compared with 7182 matched patients without diabetes, patients with diabetes had higher corticosteroid use, abdominal surgery, and drug‐related adverse events, while use of anti‐TNF and IL‐23 inhibitors was lower. These findings suggest that metabolic dysfunction may coexist with a more complicated disease course and may influence treatment patterns. Because the study was retrospective and observational, however, it cannot establish that diabetes causes more severe Crohn's disease or reduced use of advanced therapies. In the cardiovascular context, the coexistence of insulin resistance, metabolic risk factors, and chronic systemic inflammation may further increase overall cardiovascular risk, supporting closer attention to glucose control and comprehensive risk‐factor modification in IBD [47].

### 6.3 Hypertension

Unlike the association of hypertension (HTN) and CVD, the association between HTN and IBD is still unclear. In a UK‐based cohort of 2376 patients with IBD [48], it was demonstrated that IBD had a higher cumulative risk of HTN than the general population (10.9% in UC and 7.7% in Crohn's CD vs. 7.1% in non‐IBD patients, *p* < 0.001). On the other hand, Dregan et al. [43] showed that the prevalence of HTN was lower in IBD patients than in their controls. Thus, larger cohorts with better follow‐up are required to further evaluate this relationship.

### 6.4 Female Gender

Studies have shown that women with IBD have a higher risk of developing ASCVD compared to men. A systematic review by Singh et al. [49] analyzed 6478 IHD events in 123,907 patients and concluded that there is a 19% increased risk of developing IHD, especially in younger females compared to their male counterparts. Women also tend to carry a larger inflammatory burden than men, as shown by higher baseline CRP levels than men [50].

### 6.5 Obesity

While IBD patients typically have a lower BMI, obesity has been linked to a higher risk of developing IBD. Previous studies have reported that 15%–40% of IBD patients are obese and 20%–40% are overweight [51]. Obese CD patients are more likely to experience active flares, potentially due to altered drug absorption and increased inflammation. In a recent meta‐analysis of five prospective cohort studies [52], obesity was associated with an increased risk of CD (pooled HR, 1.34; 95% CI, 1.05–1.71, *I* ^2^ = 0%) compared with normal BMI. However, BMI was not associated with an increased risk of UC. Figure 3 and Table 1 show shared risk factors linking IBD with CVD.

**Figure 3:**
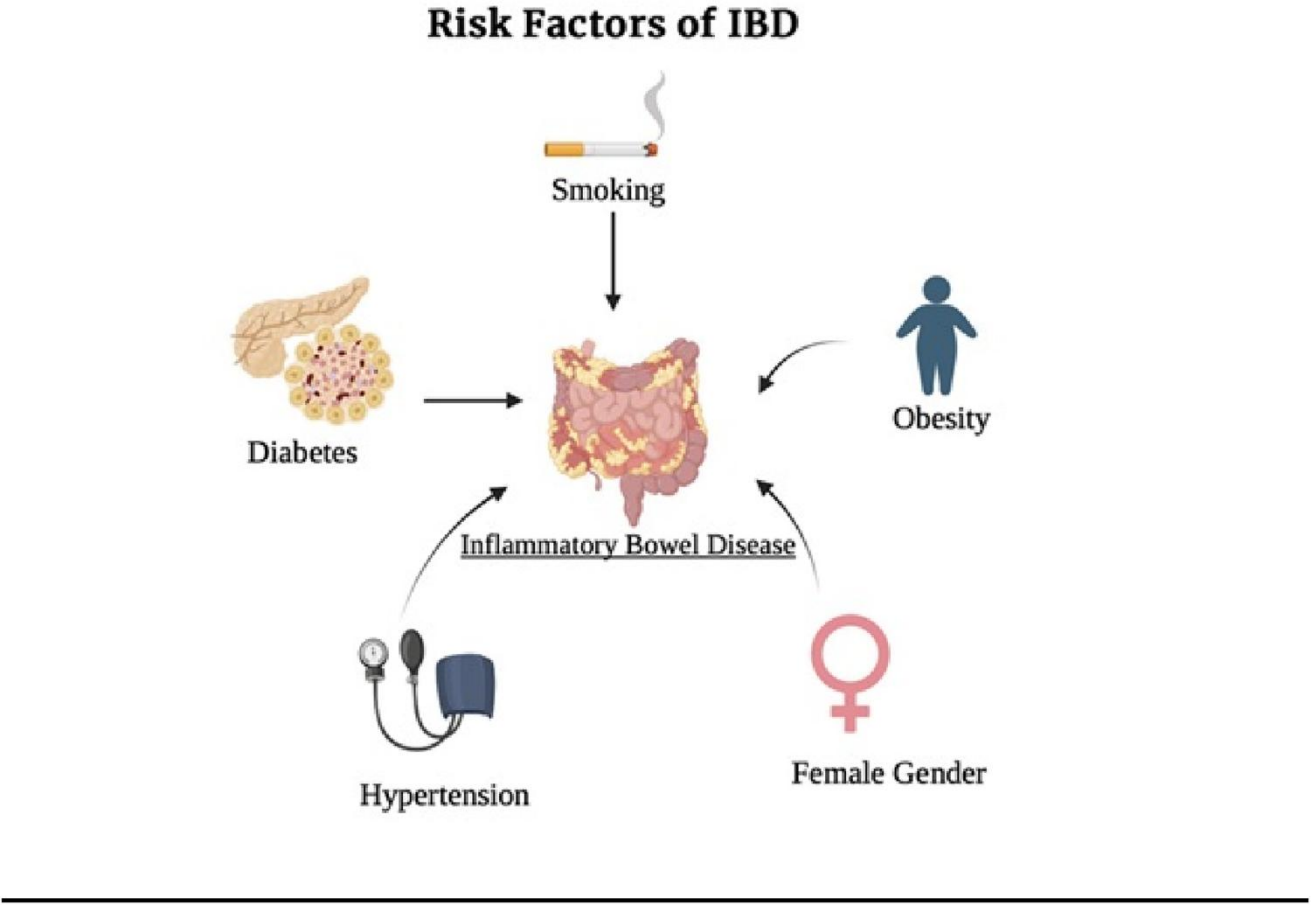
Shared risk factors linking IBD with CVD.

**Table 1:** A summary of cardiovascular manifestations associated with inflammatory bowel disease.

Cardiovascular outcome	Key findings
Coronary artery disease/myocardial infarction	Increased risk of MI, particularly during active disease
Heart failure	Increased hospitalization for heart failure
Peripheral arterial disease	1.29‐fold increased risk of PAD
Ischemic stroke	Higher risk in younger patients
Venous thromboembolism (DVT, PE)	Increased VTE risk (RR 1.96)
Atrial fibrillation	Increased AF risk during active disease
Overall ASCVD events	Higher ASCVD events (OR 2.01)

## 7 CVD Risk Assessment in IBD

The existing tools for CVD risk assessment fall short, most likely due to their failure to account for systemic inflammation or fluctuating disease activity, which would better frame the need for alternative approaches (Figure 4). The limitations are as follows:

**Figure 4:**
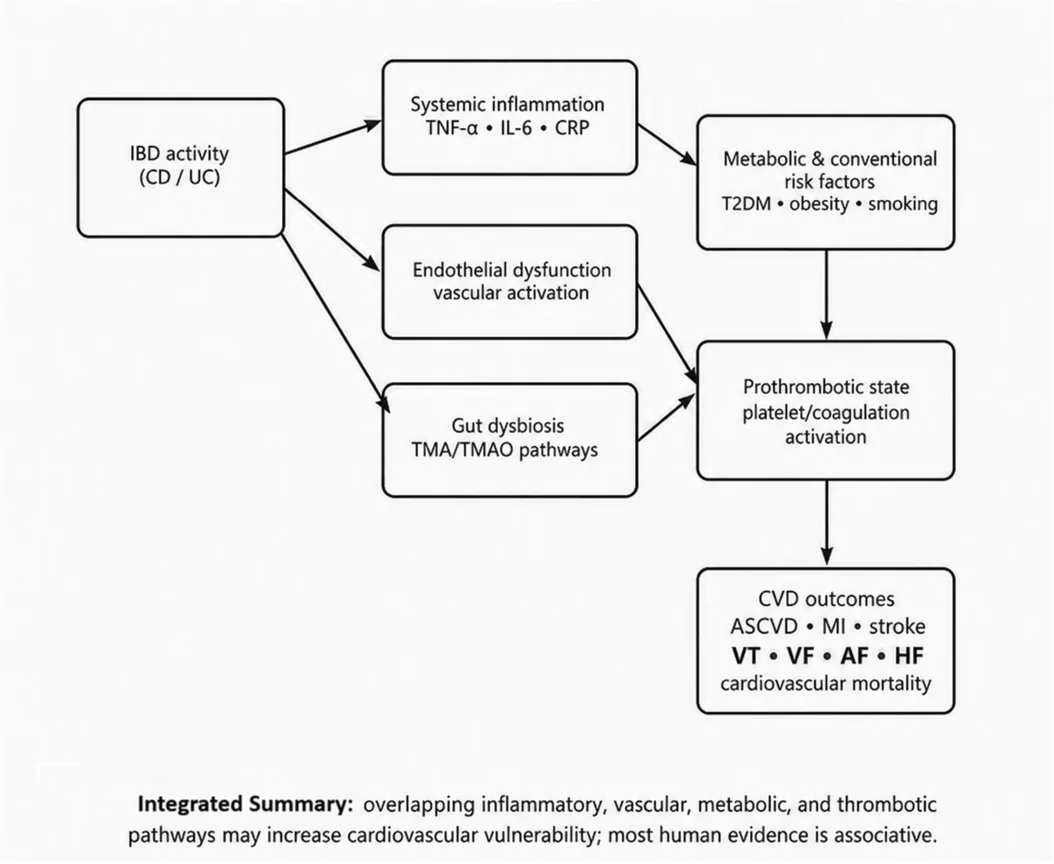
Integrated mechanistic and clinical pathway linking IBD to cardiovascular disease.

### 7.1 Limitations of Conventional Risk Assessment Tools

A dedicated assessment tool is not available for CVD risk assessment in IBD, despite a likely relation between the two. Other tools, like the AHA tool for CVD risk assessment in chronic inflammatory conditions, are often used, but the heterogeneity of classical ASCVD risk factors in IBD renders most of these classical tools insufficient [33]. The following tool can perhaps be used to better screen these patients for risk of CVD.

### 7.2 Coronary Artery Calcium Scoring

Coronary artery calcium scoring (CACS) is a non‐invasive imaging method that measures calcium deposits in coronary arteries, serving as an indicator of atherosclerosis and CAD risk [53]. Its application in individuals with IBD offers opportunities for early detection of subclinical atherosclerosis and personalized risk assessments. Integrating disease‐specific inflammatory markers and CACS can offer physicians a better understanding of the individual's risk [54]. The integration of inflammatory markers with CACS, like CRP or fecal calprotectin, may also be clinically useful. However, challenges include the lack of specific guidelines for IBD and the need to validate its predictive accuracy in this population. Further research is essential for understanding how chronic inflammation in IBD affects calcium deposition and CAD risk stratification, enabling more tailored preventive strategies [55]. While CACS provides valuable insight into subclinical atherosclerosis, it may underestimate risk in younger IBD patients who may not yet show significant calcification.

### 7.3 Screening for Disease Specific Risk Factors

Disease‐specific risk factors must be considered while making ASCVD risk assessments in patients with IBD. The most important among these are disease activity and drugs taken for IBD treatment. Studies have found patients to be at a greater risk of a cardiovascular event during an active flare or persistent disease activity of IBD as compared to during remission phases [30]. Longer disease duration in IBD patients has not been found to have a correlation with cardiovascular events, though patients have been found to be at a greater risk during the early stages of the disease [56, 57].

Corticosteroids have been found to confer a greater risk of cardiovascular events in patients taking them for IBD treatment [56, 57]. The use of 5‐aminosalicylates has been found to decrease the risk of cardiovascular events [56]. This effect might be explained by reduction in inflammation and inhibition of platelet activation by 5‐ASA. Immunomodulatory agents have not shown any significant effect on ASCVD risk [30].

## 8 Management of CVD in IBD

IBD patients, who are mostly managed by gastrointestinal specialists with an emphasis on managing acute conditions, should be screened for conventional and non‐conventional risk factors, with an awareness of increased risk for ASCVD, heart failure, and atrial fibrillation in these patients. Since the typical risk stratification tools like ASCVD risk calculation might underestimate the actual risk in these patients, it is useful to screen them for factors such as tobacco use, diabetes, and obesity that warrant an aggressive risk modification intervention. The adjunctive use of CACS alongside traditional risk assessment tools may further assist clinicians in refining cardiovascular risk estimation in this population [58].

Additionally, clinical suspicion for acute cardiac events must be kept high in patients with IBD since they have been observed to occur in patient groups who are otherwise unlikely to suffer from them for example age < 40 and females [59].

The use of cardiovascular disease–modifying drugs, for example, statins, aspirin, and clopidogrel, in IBD patients has not yet been established by guidelines, but they could prove to be of utility [60]. Patients with IBD are less likely to have lab‐established dyslipidemia, but statins, apart from their inhibition of cholesterol synthesis, have an independent anti‐inflammatory effect by inhibition of TNF‐β, interleukins, and CRP. This effect could prove to be useful in the inflammatory state that exists in IBD. Similarly, the anti‐platelet effects of aspirin and clopidogrel could be beneficial in the prothrombotic state that exists in IBD due to multiple factors discussed above.

In recent years, there has been an increase in the use of biological agents and small molecule therapies. TNF‐alpha inhibitors have shown a protective effect against arterial events by reducing systemic inflammation. Janus kinase (JAK) inhibitors, such as tofacitinib, have been used without a significant increase in MACE in broad IBD populations [61, 62]; however, they may be associated with hyperlipidemia and higher thromboembolic risk, particularly in high‐risk groups [63, 64, 65]. This highlights the need for individual baseline risk stratification before initiation [66]. Other promising drugs include IL‐12/23 inhibitors and integrin antagonists, which have demonstrated high safety profiles [67, 68]. Vedolizumab may specifically benefit patients with high baseline cardiovascular burdens. However, as some meta‐analyses suggest potential MACE risks across various advanced therapies compared to placebo, individualized surveillance remains vital [69].

## 9 Conclusion

IBD depicts a state of chronic inflammation associated with an increased risk of cardiovascular disease (CVD). While available evidence on the specific risk factors for CVD in IBD is ambiguous, proactive risk assessment and rigorous control of modifiable risk factors are suggested until future prospective research determines the clinical importance of individual contributors. Clinicians should emphasize CVD screening and prevention strategies in all IBD patients, with a focus on younger people (under 50) and women, who appear to be at a greater risk of CVD incidents.

This narrative review also has important limitations. The literature is heterogeneous, and much of the evidence linking IBD with CVD is derived from observational studies, which may be affected by residual confounding, differences in disease activity, treatment exposure, and ascertainment. The narrative design does not permit a formal quantitative synthesis or formal risk‐of‐bias assessment, and selective publication of positive associations may influence the available evidence. Accordingly, the mechanistic pathways and clinical associations discussed here should be interpreted as evidence‐supported relationships rather than proof of causality.

Future prospective studies and clinical trials are needed to clarify the impact of acute disease flares and IBD‐related therapies on the development of atherosclerotic cardiovascular disease (ASCVD), atrial fibrillation, and heart failure, further guiding evidence‐based preventive care in this high‐risk population.

## Author Contributions

**Farah Yasmin:** conceptualization, data curation, writing – original draft. **Mansoor Ahmed:** conceptualization, data curation, writing – original draft. **Taha Bin Iftikhar:** conceptualization, data curation, writing – original draft. **Asad ur Rab:** data curation, resources, writing – review and editing. **Muhammad Saeed Qazi:** investigation, methodology, project administration, writing – original draft, resources. **Muhammad Sohaib Asghar:** supervision, visualization, validation, writing – review and editing. **Mohammed Mahmmoud Fadelallah Eljack:** supervision, validation, visualization, writing – review and editing. **Krutarth Pandya:** writing – review and editing, software, validation, visualization. **M. Chadi Alraies:** supervision, visualization, validation, writing – review and editing.

## Funding

The authors have nothing to report.

## Ethics Statement

Institutional Review Board approval was not required for this study as it did not involve human subjects.

## Conflicts of Interest

The authors declare no conflicts of interest.

## Permission to Use Third Party Materials

The authors have nothing to report.

## Data Availability

The data that support the findings of this study are available from the corresponding author upon reasonable request.
